# The effect of transcutaneous auricular vagus nerve stimulation on vigilance, cognition, and mood during military exercise with acute sleep deprivation

**DOI:** 10.1186/s41235-026-00730-0

**Published:** 2026-04-13

**Authors:** Tomi Passi, Johanna Närväinen, Kristian Lukander, Jari Laarni, Mikko Lindholm, Kati Pettersson, Saija Mauno, Satu Pakarinen

**Affiliations:** 1 Finnish Institute of Occupational Health, Työterveyslaitos, P.O. Box 40, 00320 Helsinki, Finland; 2 VTT Technical Research Centre of Finland Ltd, Espoo, Finland; 3 Faculty of Social Sciences (Psychology), Tampere University, Tampere, Finland; 4 Department of Psychology, University of Jyväskylä, Jyväskylä, Finland

**Keywords:** Vagus nerve stimulation, Vigilance, Response inhibition, Mood, Sleepiness

## Abstract

Noninvasive vagus nerve stimulation provides a promising tool for supporting performance under demanding conditions, particularly during sleep deprivation. This study investigated the effects of transcutaneous auricular vagus nerve stimulation and stimulation intensity on vigilance, sustained attention, response inhibition, self-reported mood, and sleepiness in military conscripts during a 24-h training period that included total sleep deprivation. Forty-eight male conscripts completed the study. Vigilance was assessed using the psychomotor vigilance task and sustained attention and response inhibition with the sustained attention to response task. Before the stimulation, participants adjusted the stimulation intensity individually. The stimulation duration was 4 min. For the stimulation group (*n* = 24), the intensity ranged from 12 to 26 mA. In the sham group (*N* = 24), stimulation was ramped down to 0 mA during the first minute. Vigilant attention decreased with prolonged wakefulness in both groups. However, during the early morning hours at 5 am and 7 am, this impairment showed modest attenuation with increasing stimulation intensities; higher intensities were associated with smaller decrements in vigilance. No effects of stimulation were found on response inhibition. The response inhibition task is more motivationally salient than the simple vigilance task, and the impact of sleep deprivation can be obscured by strategic slowing of responses to minimize errors. Also, higher stimulation intensity was associated with a smaller shift toward negative emotional valence measured after the response inhibition task. Transcutaneous auricular vagus nerve stimulation shows potential to support vigilance during sleep deprivation in monotonous tasks, such as radar monitoring and surveillance.

**Supplementary Information:**

The online version contains supplementary material available at 10.1186/s41235-026-00730-0.

## Introduction

Non-invasive vagus nerve stimulation (nVNS) is a relatively new and promising development for supporting cognition and mood in individuals suffering from sleep deprivation. The detrimental effects of sleep loss are well-documented, including impaired alertness and emotion regulation as well as increased sleepiness (Landolt et al., 2014; Sadeghi-Bahmani et al., 2024). These deficits can result in lapsing attention, psychomotor slowing, and inhibition errors, all of which have been linked to several catastrophic accidents (Åkerstedt et al., 2011; Mitler et al., 1988). In addition, sleep loss-related functional changes in the emotional brain network connectivity often result in a low mood, increased risk-taking, and poor decision-making (Landolt et al., 2014). Such impairments can be particularly problematic for military personnel engaged in extended operational missions that require efficient, sustained mental acuity. Despite the fact that nVNS has shown promise in enhancing wakefulness and vigilance across various settings (Chen et al., 2023; McIntire et al., 2019, 2021), its effectiveness in real-world conditions, particularly in maintaining vigilant attention during sleep deprivation, remains to be explored. Moreover, while there is some evidence of the benefits of nVNS in these domains, whether taVNS has similar effects remains uncertain. The current field study aims to bridge this gap by investigating the effect of taVNS on military conscripts' vigilance, response inhibition, emotion, and sleepiness, as well as on performance in two military marksmanship tests during 24 h of total sleep deprivation. Furthermore, the effect of stimulation intensity on performance and self-reported measures will be studied.

The vagus nerve is a widely distributed primary parasympathetic nerve that connects the heart, among other internal organs, to the brainstem. Through innervation to various functionally connected brainstem nuclei, it is involved in regulating various bodily functions, such as cardiovascular activity and respiratory control, as well as sleep and alertness (Berthoud & Neuhuber, 2000; Fink et al., 2018). The vagus nerve's sensory afferent fibers ascend to the left side of the neck just above the carotid sheath, giving rise to its auricular branch extending to the proximity of the left ear. Therefore, the auricular branch can be noninvasively stimulated by delivering a low-amplitude electrical current to the cymbal conchae or the tragus of the left ear (transcutaneous auricular VNS; taVNS). Research suggests that stimulation parameters with a pulse width varying between 30 and 500 microseconds (μs) and frequencies between 7.5 and 120 Hertz (Hz) are the most effective in activating the vagus nerve and its adjacent brainstem nuclei (Hulsey et al., 2017; Yakunina et al., 2017). Depending on the aim, nVNS can be administered before, during, or after a task, potentially affecting the treatment outcome (Phillips et al., 2021). Even a single stimulation can have long-lasting effects, with benefits observed up to 19 h post-stimulation (McIntire et al., 2021).

The therapeutic effects of VNS are associated with increased activation of the brain's primary noradrenergic (NE) brainstem nucleus, the locus coeruleus (LC; Yakunina et al., 2017). The neuronal axons of the LC cover the central nervous system from the spinal cord to the cerebellum, midbrain, thalamus, and cortex (Phillips et al., 2021). The activity of the LC is positively associated with cortical arousal and wakefulness (Osorio-Forero et al., 2022). Via its NE-projections, especially to the thalamus and frontal-parietal lobes, the LC promotes arousal, attention, sensory processing, cognitive flexibility, mood, and memory, all of which are known to suffer from sleep deprivation (Lock et al., 2018; Poe et al., 2020; Samuels & Szabadi, 2008; Sara & Bouret, 2012).

While it has been shown that stimulating either the cymbal conchae or tragus activates the LC, it has been suggested that stimulation via the cymbal conchae may be more effective (Yakunina et al., 2017). This may indicate that stimulation intensity is likely one factor affecting the magnitude of LC activation; locations farther from the auricular branch may require higher stimulation intensities to achieve a comparable effect. This interpretation has also been supported by research indicating that taVNS does not activate LC when using a continuous stimulation cycle at relatively low intensities (Aranberri Ruiz, 2024), as well as research on invasive VNS showing increased activation at higher intensities (Hulsey et al., 2017). In addition, higher stimulation intensities applied to the tragus have yielded measurable effects across cortical and subcortical regions associated with the afferent vagus pathway (Badran et al., 2022). In sum, VNS activates the brainstem´s wakefulness-supporting arousal system, with higher stimulation intensities potentially leading to greater LC activation.

Due to its effects on vigilance, mood, and cognition, taVNS could be a valuable tool for supporting operational military performance, particularly under sleep deprivation. Military personnel often operate in dynamic and unpredictable operational environments in which they confront mentally, physically, and ethically challenging situations. Under such conditions, efficient information processing and vigilant attention are vital, enabling accurate detection and discrimination of targets and timely responses to environmental stimuli (Carrasco & Barbot, 2019). Strenuous military combat exercises have been demonstrated to impair cognitive functions, including vigilance, attention, memory, and reasoning, as well as result in lowered mood (Kallinen & Ojanen, 2023; Lieberman et al., 2005). Moreover, sleep loss of one night can lead to fluctuating attention, increased errors, and sluggish responses (Hudson et al., 2020; Rabat et al., 2019; Slama et al., 2018). This effect is particularly pronounced in the early morning hours after a night of wakefulness, due to circadian rhythm variations (Holding et al., 2021; Hudson et al., 2020). TaVNS in military operations is supported by its ease of use, quickness of administration, good tolerability, and long-lasting effects, which may extend from hours to even weeks (Ben‐Menachem et al., 2015).

Existing evidence on the cognitive benefits of nVNS in healthy individuals under conditions of sleep deprivation is scarce. To our knowledge, this covers only two studies (McIntire et al., 2021; Zhao et al., 2023). McIntire and colleagues (2021) suggested that brief (2 × 2-min) ctVNS on both sides of the neck supports military participants´ vigilance, multitasking, and mood under sleep deprivation for 19 h post-stimulation. In their earlier study with non-sleep-deprived individuals, they found that 2 min of ctVNS on both sides of the neck before and after a three-day cognitive testing training period improved vigilance and memory retention and that the effect lasted for 90 days post-stimulation (McIntire et al., 2019). On the other hand, Zhao and colleagues (2023) concluded that taVNS (via the cymbal conchae) improved working memory but not vigilance under sleep deprivation. The discrepancy between their findings and those of McIntire and colleagues (2021) may relate not only to the stimulation site, but also to differences in stimulation timing. Zhao et al. (2023) delivered stimulation 30 min before the cognitive testing after sleep deprivation, whereas McIntire et al. (2021) delivered stimulation in the evening before sleep deprivation. Moreover, these, along with the vast majority (if not all) of nVNS studies with healthy individuals, are conducted under stringently controlled laboratory conditions (see, for instance, Ridgewell et al., 2021), ensuring the control of extraneous factors while limiting ecological validity. We aimed to overcome this limitation by studying the effect of taVNS on cognition during conscripts´ military training. In addition, while the evidence suggests that ctVNS positively affects vigilance, taVNS (via the tragus) provides a new endeavor to extend these findings on vigilance during sleep deprivation. TaVNS could be a viable option because the auricular branch, in contrast to the cervical part of the nerve, is purely afferent, specifically targeting the brainstem (Sara & Bouret, 2012).

In this study, we examined the effect of taVNS on military conscripts’ vigilant attention and response inhibition, as well as on self-reported sleepiness and mood after one night of sleep deprivation. Vigilance was assessed using the psychomotor vigilance task (PVT), which is comparable to, for instance, radar monitoring or sentry duties and is well known for its sensitivity to detecting sleep-deprivation-related impairment in arousal (Dorrian et al., 2004). The ability to remain attentive and inhibit prepotent responses, akin to, for instance, a shooting-decision task (go/no-go), was evaluated using the sustained attention to response task (SART). We also examined the association between stimulation intensity and the magnitude of the stimulation effect on each performance measure as the conscripts adjusted the stimulation intensity individually. The following hypotheses were posed:

### H1

One night of sleep deprivation results in lengthened reaction time, impaired sustained attention, and response inhibition, accompanied by mood deterioration and increased sleepiness, all exacerbated in the early morning hours.

### H2

TaVNS supports performance, reducing the impairing effect of sleep deprivation, with increasing intensities leading to more pronounced effects.

### H3

The effects mentioned in H.2 are the most pronounced in the early morning when the pressure to sleep is the greatest.

## Methods

### Participants

**Fig. 1:**
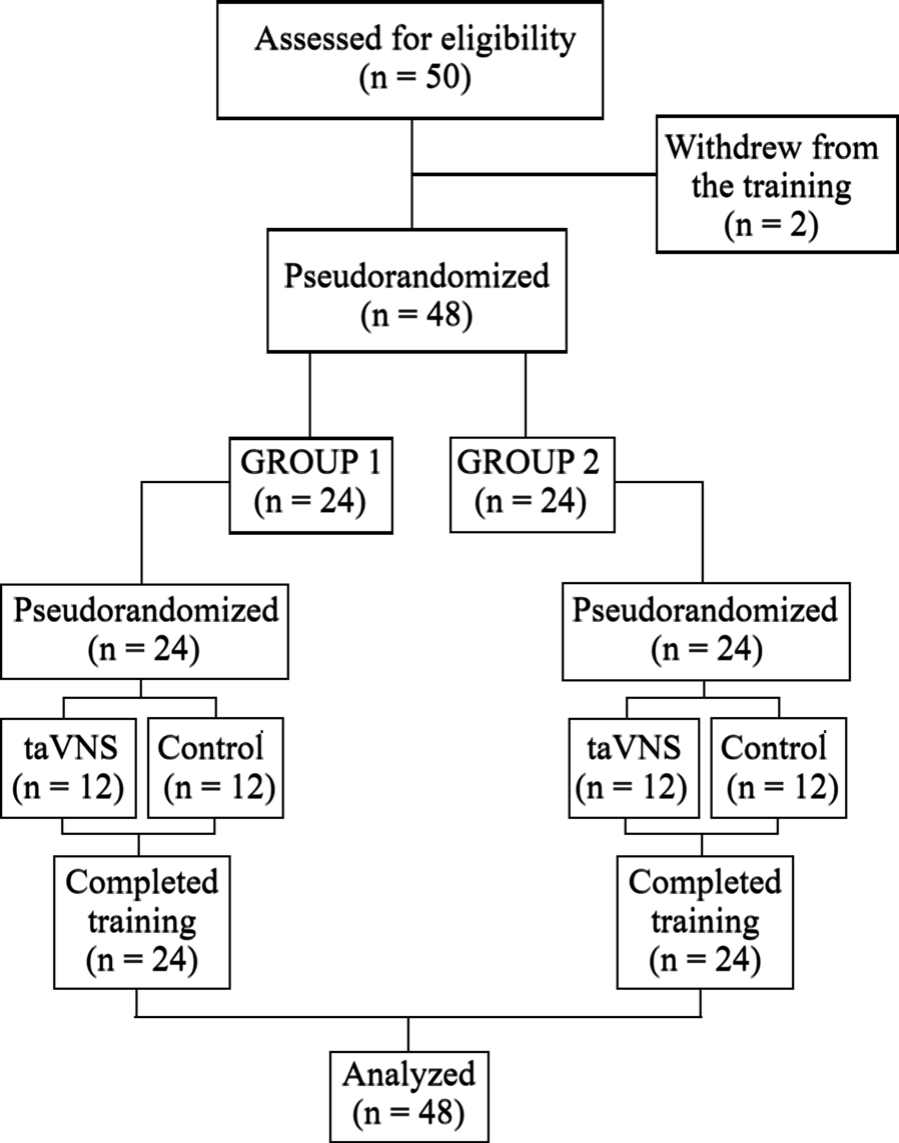
CONSORT flow diagram

The experiment was carried out as part of the Special Jaeger training program of the Finnish Border and Coast Guard Academy. The training group included 50 special border Jaeger conscripts. All volunteers (all males, *M* = 20 yrs., range: 19–25) provided their informed consent. Two participants withdrew from the study before the training. Hence, forty-eight participants completed the study (Fig. 1). The ages of the remaining 48 participants ranged between 19 and 25 (*M* = 20).

All participants had undergone extensive examinations before being allowed to join the special Jaeger forces. In addition to the requirement of passing physical fitness tests, the entrance criteria include good physical health and the absence of chronic diseases or medications. This screening was considered sufficient for this study, so no additional health information was collected in this experiment.

#### Transauricular vagus nerve stimulation and sham stimulation

The VNS stimulation was delivered via the tragus of the left ear using Nurosym (Parasym Oy, Tallinn, Estonia) VNS stimulators. These stimulators were selected because they were the only commercially available devices with a realistic, credible sham-stimulation option. The sham-stimulation devices used in previous research (MacIntire et al., 2021) targeting the cervical branch of the nerve were not available at the time the study was conducted.

**Fig. 2:**
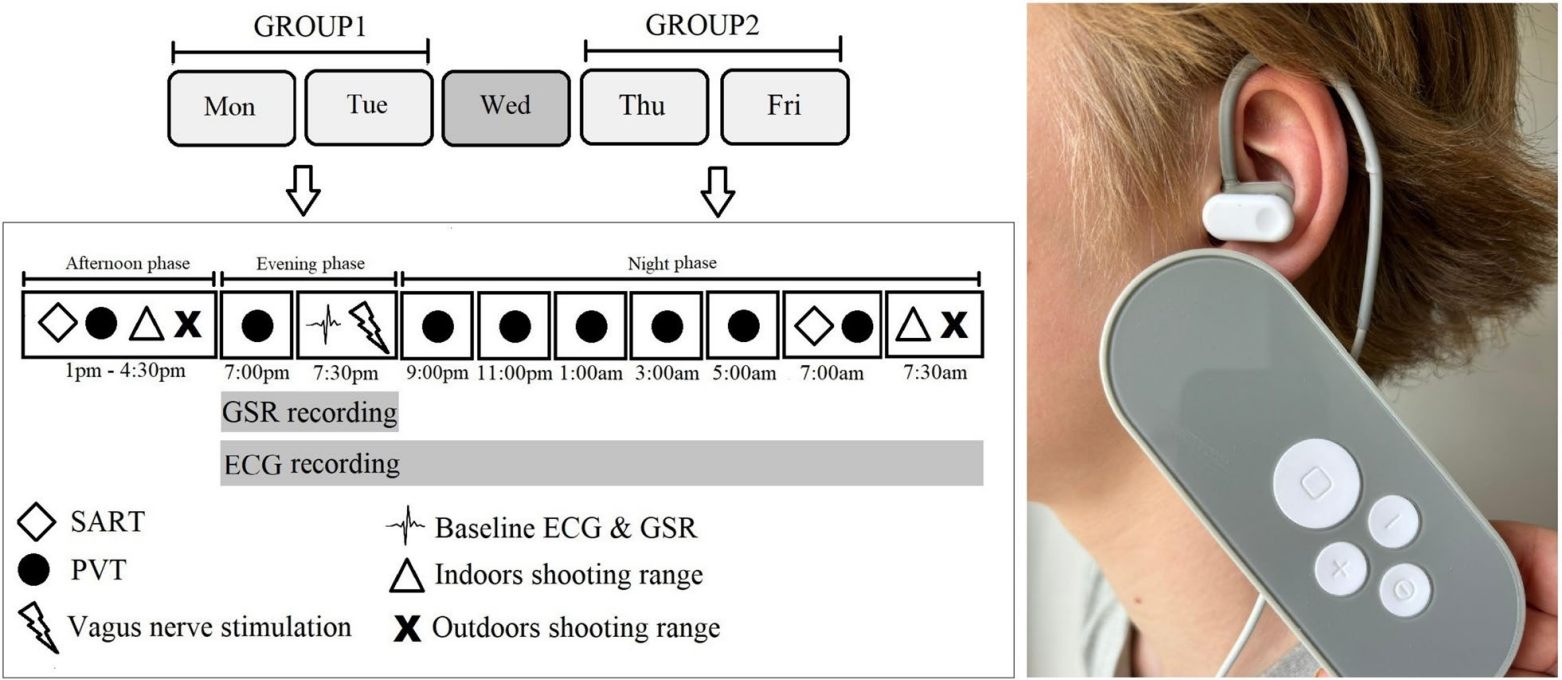
The timeline of the research activities. *Note*: The six-person teams completed the protocol in an interleaved manner. The figure on the left illustrates the timeline of research activities, showing the starting times of the first team for each task. The image on the right shows the vagus nerve stimulator used in the study

Half of the simulators were configured to deliver actual stimulation, and half were configured to deliver sham stimulation. The participants were unaware of the manipulation of the device. A numbered sticker was placed on the back of the stimulator, encoding whether it was a sham or an active device. Six devices, three in sham mode and three in active mode, were then turned face up so that the identifiers were not visible. After this, a researcher pseudorandomly distributed the devices onto separate tables in the classroom. The participants were stimulated in teams of 6 (Fig. 2) and were free to select their seats; hence, the allocation of active and sham stimulation was pseudorandomized. After the stimulation, the device identifier, together with the participant ID, was recorded on the measurement sheet to later identify which simulator was assigned to each participant. The device was attached to the tragus of the left ear with a clip. The experimenter inspected the clip to ensure it was correctly mounted and adjusted if necessary. Before the actual and sham stimulation, each participant could adjust the intensity individually. To maximize comfortable stimulation intensity, participants were instructed to increase the intensity gradually and then set it to a level that was clearly perceptible but not painful or uncomfortable. The selected stimulation intensity was noted in the measurement sheet. The other stimulation parameters were fixed: a pulse width of 200 μs, a frequency of 25 Hz, and a constant train stimulation duration of 4 min. The selection of stimulation parameters was based on earlier research suggesting neuromodulatory effects with comparable protocols (Badran et al., 2022; Ridgewell et al., 2021). In the sham stimulation condition, stimulation was initiated at the selected intensity, after which the sham devices ramped down the stimulation current to 0 mA over the first minute of stimulation. All the participants were informed that the sensation of the stimulation might change during the stimulation.

#### The sustained Attention to Response Task (SART)

In the SART, the participant's task was to react as quickly and accurately as possible to the number displayed on the tablet computer screen. They were instructed to press the space bar on a keyboard attached to the tablet device as quickly as possible when a number (1–9; GO condition) was presented on the screen, excluding number 3 (No-Go condition), in which case they had to inhibit reacting to the stimulus. Each reaction to number three was counted as an error of commission. The duration of the stimuli on the screen was 250 ms. After the stimulus presentation, a circle with an X symbol appeared on the screen for 900 ms, during which the stimulus had to be responded to or inhibited, resulting in an interstimulus interval of 1150 ms. The test duration was five minutes. Each test session contained 200 GO stimuli and 25 NoGO stimuli. The performance parameters for the task were reaction time (ms) and the relative proportion of response-inhibition errors (%). SART test parameters also include errors of omission, that is, failure to respond to a stimulus (numbers 1–9, excluding 3). In this study, we focused on errors of commission because our primary interest with the SART was response inhibition. Our motivation for choosing errors of commission was that they represent a response inhibition relevant to the shoot/not-to-shoot decision during military operations. Errors of omission were not analyzed, as they reflect a vigilance aspect of performance and were primarily assessed using the PVT.

A well-known phenomenon in the test is the so-called speed-accuracy trade-off (Dang et al., 2018; Peebles & Bothell, 2004), i.e., an increase in accuracy (a decrease in response inhibition errors) while lengthening the response time. Hence, it was emphasized that both speed and accuracy are equally important.

#### The Psychomotor Vigilance Task (PVT)

The PVT is a simple reaction time test in which the participant must react to a simple visual stimulus by pressing a key as quickly as possible. The interstimulus interval varied randomly between 1 and 9 s, and the test duration was 10 min. The median reaction time was used to represent the participant´s level of vigilance during the task. Although median reaction time has lower statistical power than transformation-based approaches, it is a robust measure of central tendency that accurately reflects typical response times within a test session (Whelan, 2008). Unlike the mean reaction time, the median is not disproportionately influenced by extreme values, making it a more reliable measure of skewed distributions of reaction time data (Whelan, 2008).

### Self-report questionnaires

#### Karolinska Sleepiness Scale (KSS)

The participants reported their perceived sleepiness using the Karolinska Sleepiness Scale (KSS; Åkerstedt & Gillberg, 1990). The KSS is a nine-point (1 = very alert, 9 = sleepy, almost falling asleep [staying awake requires effort]) sleepiness questionnaire. It was reported 18 times: after each SART and PVT test, indoor and outdoor shooting tracks, taVNS, and twice independently of any tests.

#### Three-dimensional mood questionnaire

The participants reported their moods using a three-dimensional emotional state questionnaire. The questionnaire included three subsections: the valence of the emotional state, the arousal of the emotional state, and dominance (feeling of control). In this study, only the valence of the emotional state was analyzed. Valence was assessed with a nine-point visual analog scale (VAS) (1 = sad/dissatisfied - 9 = happy/satisfied). The mood was reported 18 times: after each SART and PVT test, during indoor and outdoor shooting, taVNS, and on two additional occasions, independent of any testing.

### Procedures

The study was conducted over five days as part of the Special Border Jaeger´s marksman training (see Fig. 1). As part of the training program, 48 participants were pseudorandomly divided into two equally sized training groups (Group 1: 24 and Group 2: 24 individuals) and further into 6-person combat teams by their trainer, as determined by their training schedule. Participants performed research activities in six-person teams, progressing through the protocol sequentially. Group 1 first completed two days of marksmanship training and research-related tests in the quarters (days 1 and 2) and then, after a recovery and equipment maintenance day in mid-week, two days in the terrain without research-related activities (days 4 and 5). Group 2 followed the reverse order, beginning with the terrain training (days 1 and 2) before moving on to the marksmanship training and research activities in the quarters after the rest day (days 4 and 5). This study was conducted over two days, involving only the marksman training and tests in the quarters, i.e., no research activities were performed during the terrain training. Importantly, the participants had no training-related sleep restrictions before the experiment, regardless of the group to which they were assigned.

The training and the study procedures included three phases: an afternoon phase (between 1 and 4:30 pm), an evening phase (7:00 pm), and a night phase after the taVNS (between 9:00 pm and 7:00 am). During the afternoon phase, participants performed the SART twice and the PVT once on a tablet computer, as well as military marksmanship tests conducted at both indoor and outdoor shooting ranges. The first SART and the first PVT during the afternoon phase were conducted to familiarize participants with the tests and to minimize practice effects that could confound the results, especially with the SART. The second SART conducted during the afternoon phase was included as a baseline measure. After the afternoon phase, there was a break of approximately 2.5 h before the evening and night phases.

At the beginning of the evening phase, the participants were equipped with Bittium Faros 360 heart rate monitors (Bittium Corporation, Oulu, Finland) and Shimmer3 skin conductance devices (Shimmer Research Ltd, Dublin, Ireland), after which they underwent the PVT baseline test in a classroom. This was followed in another classroom by four minutes of baseline skin conductance and heart rate measurements, followed by four minutes of taVNS or sham stimulation. After the taVNS, the PVT was administered six additional times at 2-h intervals during the night phase (i.e., a total of 7 times; Fig. 2). The duration of one PVT test for the entire group during the night phase was one hour. Hence, circadian rhythm variations in PVT performance were expected to be minimized across the participants.

The SART and indoor and outdoor marksmanship tests were conducted once after the taVNS on the following morning, after sleep deprivation (the night phase; Fig. 2). After each test, the participants evaluated their sleepiness with the KSS, their emotional valence and arousal with the three-dimensional emotion questionnaire, and their workload with the NASA Task Load Index (NASA-TLX; Hart & Staveland, 1988). The NASA-TLX, electrocardiography (ECG) and galvanic skin response (GSR) recordings, and the military marksmanship test results are not reported here. After the last test, the participants returned their heart rate monitoring devices.

The participants were not allowed to sleep during the night of the experiment. They were instructed to march and parry the garrison's barracks area. The participants were equipped with ActiGraph GT9X Link move sensors (ActiGraph LLC, Pensacola, FL, USA) to monitor their activity throughout the night, ensuring they remained active and did not have rest periods.

### Statistical analyses

#### Transauricular vagus nerve stimulation (taVNS)

To examine stimulation intensity (mA), the mean and median will be calculated.

#### Cognitive testing

For each statistical analysis, R version 4.3.1 (R Core Team, 2023) was used. We conducted three separate linear mixed models on each response variable (*PVT median reaction time for hits*, *SART commission errors*, and *SART mean reaction time for correct hits*) using the *lme* function from the *nlme* package (Pinheiro & Bates, 2023). Regarding each response variable, the primary model consisted of a test session (sessions 1–7 in PVT and 1–2 in SART), stimulation intensity (mA), and their interaction as fixed effects. The participant was set as a random effect (random intercept model). The significance of the fixed effects was assessed using F-tests obtained from the anova() function of the *stats* package. When the interaction was not significant, the model results were additionally evaluated without the interaction term. We also assessed residual heterogeneity by fitting different variance structures using variance functions available in the *nlme* package. The fit of these models with different variance structures was compared using the Akaike Information Criterion (AIC), Bayesian Information Criterion (BIC), and the Likelihood ratio test (L.Ratio), with significance determined at the α-level of 0.05. Last, partial eta squared (*ηp*^*2*^) values for F-tests were calculated using the *effectsize* package (Ben-Shachar et al., 2020). Effect sizes were interpreted according to the guidelines suggested by Cohen (1988). 

#### Self-report questionnaires

Since the SART and PVT differ in their cognitive demands and durations, the levels of sleepiness and emotional valence were examined separately with both tests. Considering the ordinal scale of the KSS and emotional valence data, a cumulative link mixed-effects model was used for the analysis, in accordance with the recommendations (Taylor et al., 2023). The *clmm* function of the *ordinal* package was used in the analysis (Christiansen, 2022). The model specification and testing of the fixed effects followed the procedure described in section Cognitive testing, excluding variance structure modeling, which is not applicable to cumulative link mixed-effects models. The participant was set as the random variable.

## Results

### Transauricular vagus nerve stimulation (TaVNS)

The self-administered stimulation intensity among participants varied between 12 and 26 mA. The average intensity was 19.6 mA (*SD* = 4.26), and the median intensity was 20 mA.

### The Psychomotor Vigilance Task (PVT)

Initially, the interaction between the PVT test session and the stimulation intensity was not significant (*F*[6, 276] = 1.93, *p* = .076). However, residual diagnostics indicated non-constant variance: reaction-time residuals broadened over the night (cone-shaped heteroscedasticity across sessions), and residual variability also varied with stimulation intensity. To address this, we applied different variance functions to improve the model fit. The results of the model comparisons are in Table 1. The best-fitting model used the *varComb* function in the *lme* package, allowing differing residual variances by test session via varIdent () and changing residual variance as a function of a continuous predictor stimulation intensity via varExp().

**Table 1:** Model comparisons for various variance structures in the linear mixed-effects models

Model	df	AIC	BIC	L.Ratio	L.ratio vs. previous	*p*
1	16	4209.66	4270.05	− 2088.83	–	–
2	22	3733.87	3816.91	− 1844.94	487.78	*p* < .001
3	23	3694.05	3780.86	− 1824.02	41.83	*p* < .001
4	23	3676.44	3763.25	− 1815.22	–	–

Variance structures for the fitted models were as follows: Model 1, homogeneous residual variance; Model 2, varIdent by PVT test session; Model 3, varIdent by PVT test session plus varIdent by taVNS group; Model 4, varIdent by PVT test session plus varExp as a function of stimulation intensity

The results of the best fit model (Model 4) showed that both the PVT test session (*F*[6, 276] = 18.67, *p* < .001; *ηp*^*2*^ = 0.29, 95% CI [0.21, 1.00], large effect) and the interaction between the test session and the taVNS group (*F*[6, 276] = 2.49, *p* = .023; *ηp*^*2*^ = 0.05, 95% CI [0.00, 1.00], small effect) were statistically significant predictors of median reaction time.

**Fig. 3:**
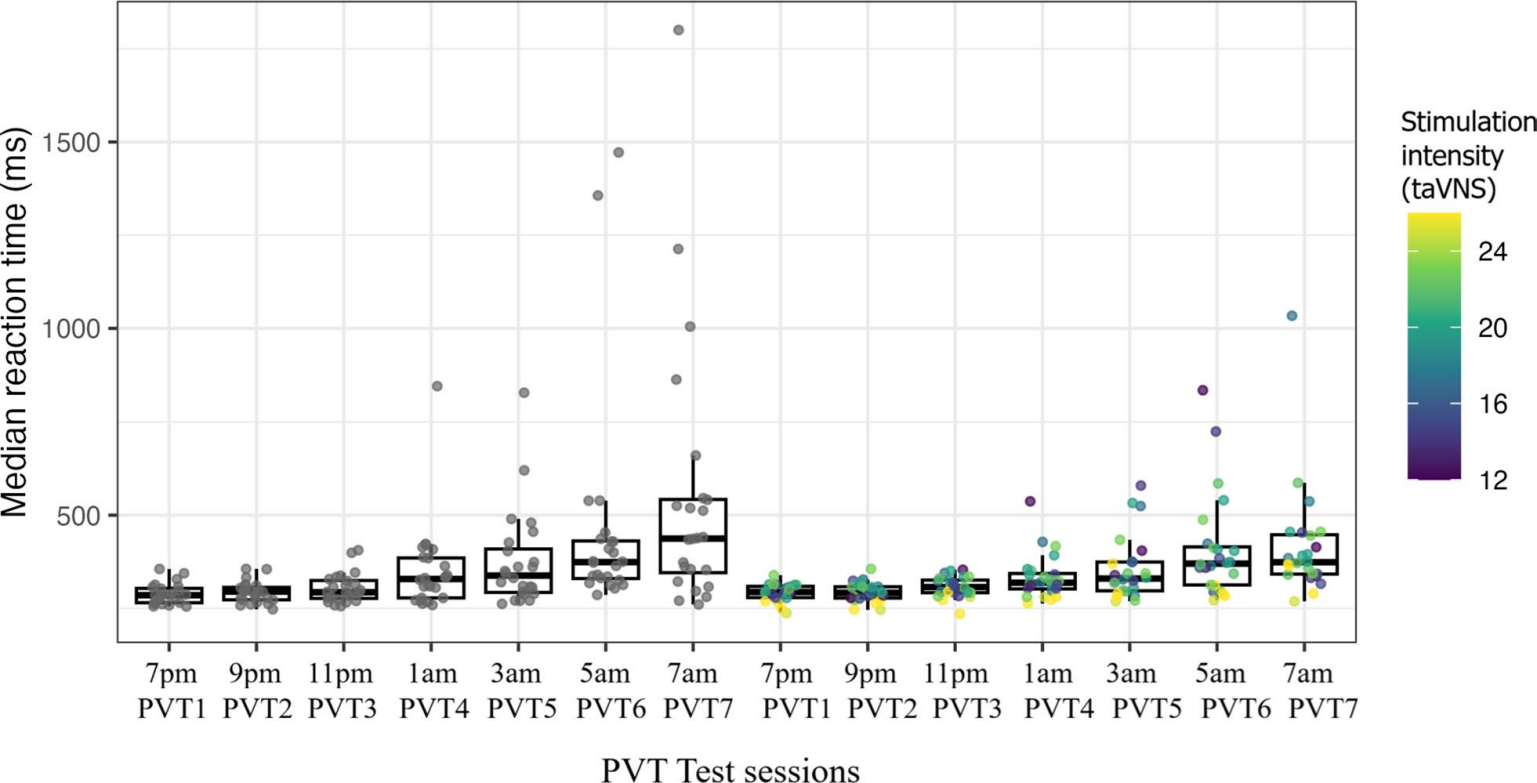
Observed PVT median reaction times by taVNS group.* Note*: The observed median reaction time (ms) (y-axis) of the taVNS (right-side box plots) and sham group (left-side box plots) participants during the seven PVT test sessions (x-axis). Participants received either active taVNS stimulation or sham stimulation after the first PVT test session between 7:30 and 8:30 pm. The box depicts 50% of the values (the interquartile range, IQR), excluding the top and bottom 25%. The top and bottom of the whiskers represent the minimum and maximum, excluding outliers (defined as 1.5 × IQR). The horizontal line in the box represents the median value

The main effect of the PVT test session indicated that the median reaction times significantly increased in sessions 3 to 7 compared to the baseline (see Table 2 for exact values). However, the interaction term suggested that stimulation had a statistically significant effect on median reaction time in the sixth and seventh PVT test sessions (see Table 2 for exact values). Observed reaction time values are shown in Fig. 3.

**Table 2:** Linear mixed effects model estimates for the PVT median reaction time

Fixed effect	*B* (ms)	*SE*	95% CI
PVT2	4.98	4.59	[− 4.05, 14.01]
PVT3	19.28**	7.08	[5.35, 33.21]
PVT4	71.06***	18.66	[34.32, 107.81]
PVT5	98.92***	26.18	[47.38, 150.46]
PVT6	202.59***	49.35	[105.44, 299.75]
PVT7	257.80***	59.53	[140.62, 374.98]
stim.intens	0.12	0.38	[− 0.65, 0.88]
PVT2 X stim.intens	− 0.21	0.24	[− 0.68, 0.25]
PVT3 X stim.intens	− 0.36	0.36	[− 1.08, 0.36]
PVT4 X stim.intens	− 1.77	0.96	[− 3.66, 0.12]
PVT5 X stim.intens	− 2.18	1.35	[− 4.83, 0.48]
PVT6 X stim.intens	− 5.19*	2.54	[− 10.19, − 0.19]
PVT7 X stim.intens	− 6.74*	3.07	[− 12.77, − 0.70]

Significance: *p* < .05*; *p* < . 01**, *p* < . 0001***; PVT X stim.ins = PVT test session and stimulation intensity interaction

In summary, while participants' median reaction times increased from the third PVT test session onward, higher stimulation intensity was associated with shorter median reaction times on the last two testing sessions, at 5 am and 7 am.

### The Sustained Attention to Response Task (SART)

No significant main effects of the test session and stimulation intensity, or their interaction, were found on commission errors, which remained stable over both SART tests for both groups. Concerning the SART reaction time, neither the interaction between the test session and stimulation intensity nor the main effect of stimulation intensity was a significant predictor in the model. Therefore, only the model with one significant fixed effect, the test session, was investigated. The test session was a significant predictor of the mean reaction time (*F*[1, 47] = 23.30, *p* < .001, *ηp*^*2*^ = 0.33, 95% CI [0.16, 1.00], large effect). The model indicated that participants' mean reaction time to go stimuli lengthened (*B* = 54.19 ms, SE = 11.23, 95% CI = [31.61, 76.78]) in the second testing after one night of wakefulness compared to the first testing.

### Emotional valence after the PVT

Neither the interaction between the test session and stimulation intensity nor the main effect of stimulation intensity was a significant predictor in the model. The simple model with only one significant fixed effect, the test session, was investigated. The results showed that the probability of experiencing sadness/dissatisfaction increased significantly after the third (*β* = − 0.44, SE = 0.22, 95% CI [− 0.86, − 0.01]), fourth (*β* = − 0.56, SE = 0.22, 95% CI [− 0.98, − 0.13]), fifth (β = − 0.79, SE = 0.22, 95% CI [− 1.22, − 0.36]), sixth (*β* = − 1.11, SE = 0.22, 95% CI [− 1.55, − 0.68]), and seventh (*β* = − 1.18, SE = 0.22, 95% CI [− 1.62, − 0.74) PVT test sessions compared to the first. The threshold coefficients of the model for the emotional valence scale categories are reported in Supplementary Table S1.

### Emotional valence after the SART

In the full model, each fixed effect, the test session, stimulation intensity, and their interaction, were found to be significant predictors of emotional valence measured after the SART test. The results showed that the probability of experiencing sadness/dissatisfaction increased statistically significantly (*B* = − 2.94, SE = 0.68, 95% CI [− 4.27, − 1.61]) after the morning SART test compared to the emotion questionnaire conducted after the first SART test. Also, a significant main effect of the stimulation intensity was found (*B* = − 0.10, SE = 0.04, 95% CI [− 0.18, − 0.01]); however, this was not further interpreted in the presence of a significant test session and stimulation intensity interaction. Significant interaction (*B* = 0.10, SE = 0.04, 95% CI [0.02, 0.18]) indicated that the change in emotional valence from baseline to the post-sleep-deprived state depended on stimulation intensity. Specifically, the higher stimulation intensity was associated with a reduced shift toward negative emotional valence over the two time points. The threshold coefficients of the model for the emotional valence scale categories are reported in Supplementary Table S2.

### Sleepiness after the PVT

Interaction between the test session and stimulation intensity was not a significant predictor in the model. The simple model with only one significant fixed effect, the test session, was investigated. The results showed that the probability of experiencing sleepiness increased significantly after the second (*β* = 0.95, SE = 0.39, 95% CI [0.20, 1.71]), third (*β* = 2.67, SE = 0.41), 95% CI [1.86, 3.47]), fourth (*β* = 4.40, SE = 0.46, 95% CI [3.50, 5.29]), fifth (*β* = 5.74, SE = 0.50, 95% CI [4.75, 6.73]), sixth (*β* = 7.19, SE = 0.56, 95% CI [6.10, 8.28]), and seventh (*β* = 6.38, SE = 0.53, 95% CI [5.34, 7.42) PVT testing sessions compared to the sleepiness after the first PVT. The threshold coefficients of the model for the sleepiness scale categories are reported in Supplementary Table S3.

### Sleepiness after the SART

Sleepiness was also examined after the SART. A random structure investigation revealed near-zero variance across participants, indicating insufficient participant-level variability. Therefore, we proceeded with a cumulative link model (CLM) without random effects using the *clm* function in the *ordinal* package [0]. The interaction between test session and stimulation intensity showed only a weak trend (*B* = − 0.07, SE = 0.04, 95% CI [− 0.14, 0.00]), and the stimulation intensity main effect was not a significant predictor in the model. Hence, the final model included only one significant predictor, the test session. The probability of experiencing sleepiness increased significantly after the morning SART test compared to the probability of sleepiness measured before sleep deprivation (*B* = 3.26, SE = 0.49, 95% CI [2.33, 4.27]). The threshold coefficients of the model for the sleepiness scale categories are reported in Supplementary Table S4.

## Discussion

Research investigating nVNS in healthy sleep-deprived individuals is scarce, and the existing studies have been conducted in a laboratory setting (McIntire et al., 2021; Zhao et al., 2023). The current study was the first to examine the effect of brief 4-min taVNS, delivered to the tragus, on cognition, mood, and sleepiness under sleep deprivation in a field experiment. In accordance with H.1, overnight sleep deprivation impaired cognitive performance, as indicated by the reduced psychomotor speed in the simple reaction time test (PVT) and the go-nogo test (SART). Similarly, in line with H.2 and H.3, vigilance maintenance was supported by taVNS in sleep-deprived individuals in the early morning, as measured with the simple reaction time test. Our findings contribute to previous research (McIntire et al., 2021) by suggesting that, similar to ctVNS—although not as strongly—taVNS is associated with vigilance maintenance during sleep deprivation, and this effect can be observed in a more ecologically valid setting. The current study provides preliminary evidence for an association between stimulation intensity and vigilance, such that higher intensities are associated with better psychomotor performance during the highest pressure to sleep, i.e., early morning hours, in sleep-deprived individuals. Lastly, higher taVNS stimulation intensity was associated with a smaller shift toward negative mood valence after a sleepless night, as measured after the go-nogo test. It is important to note that taVNS was related to reduced negative effects of sleep deprivation on vigilance and mood; however, based on the present findings, it cannot be concluded whether taVNS would influence performance or mood in the absence of sleep deprivation or other performance-impairing factors. Overall, the results of the present study indicate that a single taVNS session is modestly associated with vigilance maintenance, with the effect lasting overnight up to 12 h post-stimulation.

PVT findings align with the predictions of well-established alertness regulation models, particularly the Three-Process Model of Arousal Regulation proposed by Åkerstedt and Folkard (1997). According to the model, prolonged wakefulness increases homeostatic sleep pressure (process S), whereas the circadian rhythm component (process C) follows a 24-h sinusoidal pattern, reaching a nadir in wakefulness in the early morning. These processes together result in a vigilance performance peak in the early evening, followed by a gradual decrement, with the most pronounced, critical loss of vigilance observed in the early morning. Our findings on PVT reaction time are consistent with this trajectory. The most stable performance was observed in the evening at 7 pm, followed by a decline beginning around midnight, with the greatest psychomotor performance impairments in the morning at 5 and 7 am. The findings indicate that taVNS was modestly associated with vigilance maintenance when, due to the combined effects of S and C, alertness is predicted to fall below a critical level—a level characterized by excessive sleepiness and marked performance impairment (Åkerstedt & Folkard, 1997). During this critical state, the main driver of homeostatic sleep pressure, extracellular adenosine buildup in the brain, inhibits arousal system activation, and circadian rhythmic variation reaches its trough. Taken together, taVNS may be most effective in the early morning hours under a typical 24-h sleep-deprivation schedule.

While this interpretation may be applicable to the waking schedule used in the current study, it may limit further generalization to the military operations encountered in the real world. Such operations often extend over multiple days and involve chronic sleep restriction. This can result in circadian misalignment and shifts in circadian rhythmicity (McEwen & Karatsoreos, 2015), making it more difficult to predict the optimal stimulation window. This timing uncertainty—coupled with wake-time-dependent increase in homeostatic sleep pressure and its major recovery occurring in the first hours of sleep (Åkerstedt & Folkard, 1997)—underscores the need for future studies to investigate the effectiveness of nVNS applications during prolonged operations involving chronic partial sleep restriction.

The PVT is highly sensitive to the impairing effects of sleep loss due to its monotonous, mentally fatiguing nature (Dorrian et al., 2004). The test requires participants to maintain a top-down regulated alert state, which enables them to detect and respond to targets rapidly. In our study, higher stimulation intensities were associated with a reduced lengthening in reaction time during early-morning test sessions at 5 am and 7 am. This pattern suggests that higher stimulation intensities may recruit vagus nerve fibers more effectively than lower intensities, potentially including non-myelinated C-fibers. The observed dose-dependent relationship may indicate that higher stimulation intensities could lead to higher extracellular NE concentrations in frontal cortical regions. However, no physiological biomarkers were collected, and hence, this assumption remains speculative. Future studies combining stimulation with neuroimaging measures would be required to investigate the mechanisms by which stimulation intensity modulates psychomotor performance.

TaVNS stimulation intensity also appeared to be related to between-participant reaction time variability. In the sham group, we observed a pronounced increase in between-participants reaction time variance as the experiment progressed overnight; as sleep debt accumulated, some individuals maintained high performance, whereas others showed a substantial lengthening of reaction time (see Fig. 3). This increased between-participant variation likely reflects differential vulnerability to the adverse effects of sleep loss. In contrast, in the taVNS group, the increase in reaction time variability was more modest (see Fig. 3), suggesting that higher stimulation intensities were linked to less pronounced performance decrements in sleep‑loss‑vulnerable individuals. Future studies investigating cognitive performance under sleep deprivation should examine how individual characteristics associated with sleep loss vulnerability (e.g., baseline reaction time variability; Chua et al., 2019) modulate the effectiveness of VNS. This would not only help us better understand whether VNS primarily benefits those whose cognitive performance suffers most from sleep loss but could also provide more precise estimates of stimulation effects, hence improving statistical power to detect group differences.

Not all research evidence on nVNS in sleep deprivation is uniform. The somewhat conflicting findings may be attributable to differences in experimental conditions, such as the timing of stimulation. Zhao and colleagues (2023), in a comparable military setting, reported effects of taVNS only on working memory, with no effects on vigilance. In their study (Zhao et al., 2023), however, the stimulation was administered after a night of wakefulness, immediately before cognitive testing. In contrast, in our study and in that of McIntire and colleagues (2021), the stimulation was delivered in the evening before the sleep deprivation period. While it is well established that stimulation delivered shortly before testing can acutely enhance executive functioning (Colzato & Beste, 2020), longer-term neuromodulatory effects on vigilance may be more effectively achieved by administering stimulation well in advance of sleep deprivation, for example, on the preceding evening (Olsen et al., 2023). Hence, the temporal relationship between stimulation and a cognitive task is likely one factor to determine which cognitive domain is affected.

Response inhibition, as indexed by errors of commission on the SART, remained intact after a night of sleep deprivation, while response times increased. This aligns with a previous study involving sleep deprivation in a comparative military context (Passi et al., 2022). Two factors may account for this phenomenon: stimulating task characteristics and strategic slowing in response speed. First, SART provides immediate feedback on the screen when an error of commission is made, which is shown to enhance performance under sleep deprivation (Massar et al., 2019). This effect is akin to the impact of VNS—both feedback and VNS are associated with increased activation of the LC-NE system, resulting in improved cognitive performance. It is also noteworthy that the SART, unlike the PVT, was administered only once during the sleep deprivation period. Hence, it may have introduced novelty—a factor known to increase NE signaling (Bellesi et al., 2016). Second, lengthening of response times may reflect a resource-conserving responding strategy, where accuracy is prioritized over speed. Avoiding errors may have become a more salient performance indicator, encouraging slower but more controlled responding (see, for instance, Dang et al., 2018). Future studies could tailor cognitive tests to better assess the effects of altered arousal states on cognition. For instance, omitting error feedback from the task might allow one to study both response inhibition and error awareness (Brosnan et al., 2018)—a phenomenon linked to the LC-NE system functioning (Sellaro et al., 2015). Lastly, administering more stimulating tasks multiple times could make them feel more routine-like, thereby reducing the novelty effect when assessing performance on well-learned tasks.

In military contexts, some tasks demand simple signal detection and rapid, overlearned responding, whereas others combine vigilance with decision-making and require sustained, goal-directed monitoring and higher-order cognitive control for infrequent but critical stimuli (Wohleber et al., 2019). Although laboratory studies suggest that taVNS can influence higher-order cognition (Ridgewell et al., 2021; Su et al., 2025), in the current study, stimulation intensity was associated only with vigilance maintenance, as measured by the PVT—a simple vigilance task that relies on well-learned, habituated responding. In contrast, no effect on the SART—a test that requires higher-order cognitive control—was observed. As mentioned earlier, the latter may have resulted from the task's novelty effect, error feedback, or the speed-accuracy trade-off effect. In future, studies should provide further clarification on whether taVNS could support higher-order decision-making and cognitive control in more realistic operational settings outside the laboratory.

Higher stimulation intensity was associated with a smaller shift in mood toward negative valence, as measured after SART but not after PVT. Similarly, there was a weak trend suggesting that taVNS may have reduced self-reported sleepiness after the SART, but not after the PVT. One possible explanation for the task-specific effect is that the SART was 5 min shorter and performed only twice, and thus was more engaging and elicited a sense of novelty, compared to the 10-min PVT, which was performed 7 times overnight. Furthermore, the PVT is more monotonous than the SART and can therefore be considered more mentally demanding. In low-arousal states, novelty is known to increase physiological arousal (Coull, 1998), which may have had an additive effect on arousal with taVNS and, therefore, contributed to the smaller decrease in emotional valence. The PVT, in contrast, might have led to equally high levels of perceived boredom and sleepiness among all participants. However, this interpretation should be considered tentative due to the lack of robust between-group differences in sleepiness after the SART.

Six limitations were concluded from the study. First, the participants were relatively young, fit males, which may limit the generalizability of the results to the broader population. Second, the sample sizes in the two groups were relatively small, which added uncertainty to the model estimates, as reflected in the wide confidence intervals. These results may warrant reproduction with a larger sample size to improve the reliability of the findings. Third, since the SART was not administered repeatedly, our understanding of how taVNS affects response inhibition during sleep deprivation in well-learned, routinized real-life tasks is limited. Fourth, we did not collect sleep data from the night preceding the experiment and therefore cannot evaluate the effects of possible between-participant differences in the quantity or quality of sleep before the prolonged wakefulness. However, because participants were pseudorandomly assigned to the stimulation and sham groups prior to the experiment, this variability is expected to be balanced between the two groups. Fifth, we did not include a formal manipulation check assessing participants’ beliefs about whether they received active or sham stimulation; however, informal post-study interviews indicated that participants were unaware of the sham manipulation. Finally, given the wide confidence intervals of the partial eta squared estimates—especially for the stimulation intensity-related effect—they should be interpreted with caution. The uncertainty in these estimates reflects the dominant contribution of test-session-level variance, the emergence of stimulation-related effects only in the early-morning test sessions, and the inherent variability of the linear mixed-effects models. For these reasons, effect sizes are reported for transparency and completeness of the statistical analysis rather than exact estimates.

### Practical implications

Sleep deprivation results in a low arousal state, as evidenced by psychomotor slowing during a monotonous vigilance task. However, as indicated by our study, taVNS was modestly associated with smaller declines in alertness under conditions of monotony and limited external stimulation. Hence, taVNS shows some promise for supporting performance in both military and civilian contexts, where maintaining high performance is crucial, and where monotony, long working hours, and night shifts are common, such as radar monitoring, vehicle transportation, sentry duties, nuclear power, healthcare, aviation, and rail and road transportation. In such environments, protecting the ability to rapidly detect critical environmental changes and to support vigilant responses to those events is essential. In addition, taVNS might offer a potential alternative to traditional stimulants, such as caffeine and nicotine, as their effect is likely more short-lived, and their impact on cognition is attenuated by chronic use (Miller et al., 2018). TaVNS is quick and easy to administer and may suit a wider variety of people as the auricular branch is solely afferent, i.e., it does not innervate the heart.

## Conclusion

The current study demonstrated that sleep deprivation impaired cognitive performance on a monotonous cognitive task, with the most pronounced effects observed at 7 am, the early morning after a night of wakefulness. The most significant finding was that higher taVNS stimulation intensity was modestly associated with a reduced vigilance decrement at 5 and 7 am during the monotonous task. Conversely, this was not the case with a more engaging go-nogo cognitive test, in which the test's stimulating (i.e., motivational) nature likely obscured the effect. Higher taVNS stimulation intensity was also associated with reduced shift in mood toward negative valence. These effects were obtained from tests conducted in a classroom setting during conscripts' military field training, yielding greater ecological validity than studies conducted under strict laboratory conditions. TaVNS may have practical relevance in mitigating vigilance decrement during routine-like activities when the task itself provides little stimulation under conditions of sleep deprivation.

## Supplementary Information

Additional file1 (DOCX 19 KB)

## Data Availability

The participants have not given their consent to publish their data. However, the data can be made available for inspection if requested. Requests to access these datasets should be directed tomi.passi@ttl.fi and satu.pakarinen@ttl.fi.

## Abbreviations

GSR: Galvanic skin response
ctVNS: Cervical transcutaneous vagus nerve stimulation
ECG: Electrocardiography
KSS: Karolinska Sleepiness Scale
LC: Locus coeruleus
NT: Nucleus tractus solitarius
PVT: Psychomotor vigilance tasks
SART: Sustained Attention to Response Task
taVNS: Trans auricular vagus nerve stimulation
NASA-TLX: NASA Task Load Index
nVNS: Non-invasive vagus nerve stimulation
VAS: Visual analog scale
VNS: Vagus nerve stimulation
