# Supplementary material for: The effect of transcutaneous auricular vagus nerve stimulation on vigilance, cognition, and mood during military exercise with acute sleep deprivation
Source: Cogn Res Princ Implic. 2026 Apr 13;11:32. doi: 10.1186/s41235-026-00730-0 (PMC13076714; doi:10.1186/s41235-026-00730-0)
Supplement: Supplementary file 1 — Additional file1 (DOCX 19 KB) [file 41235_2026_730_MOESM1_ESM.docx]

**Supplementary material**

**Table S1**.

*The Threshold Coefficients for Valence Ratings Associated with the PVT*

| Valence scale | Estimate | 95% CI |
| --- | --- | --- |
| 1\|2 | -4.47 | -5.29 – -3.64 |
| 2\|3 | -3.76 | -4.41 – -3.11 |
| 3\|4 | -3.26 | -3.86 – -2.66 |
| 4\|5 | -2.35 | -2.89 – -1.81 |
| 5\|6 | -0.84 | -1.35 – -0.35 |
| 6\|7 | 0.14 | -0.35 – 0.64 |
| 7\|8 | 1.30 | 0.79 — 1.82 |
| 8\|9 | 2.56 | 1.96 — 3.16 |

**Table S2.**

*The Threshold Coefficients for Valence Ratings Associated with the SART*

| Valence scale | Estimate | 95 % CI |
| --- | --- | --- |
| 2\|3 | -8.10 | -10.73 – -5.47 |
| 3\|4 | -6.63 | -8.82 – -4.44 |
| 4\|5 | -5.11 | -7.00 – -3.23 |
| 5\|6 | -2.49 | -3.97 – -1.02 |
| 6\|7 | -1.19 | -2.53 – 0.15 |
| 7\|8 | 1.76 | 0.34 – 3.18 |
| 8\|9 | 6.19 | 2.99– 9.40 |

**Table S3.**

*Threshold Coefficients for Sleepiness Ratings Associated with the PVT*

| Threshold coefficients | | |
| --- | --- | --- |
| KSS scale | Estimate | 95% CI |
| 1\|2 | -3.58 | -4.72 – -2.45 |
| 2\|3 | -2.40 | -3.30 – -1.51 |
| 3\|4 | -0.43 | -1.20 – 0.33 |
| 4\|5 | 1.04 | 0.27 – 1.82 |
| 5\|6 | 2.54 | 1.71 – 3.36 |
| 6\|7 | 4.21 | 3.30 – 5.11 |
| 7\|8 | 5.74 | 4.75 – 6.73 |
| 8\|9 | 8.23 | 7.08 – 9.39 |

**Table S4.**

*The Threshold Coefficients for Sleepiness Ratings Associated with the SART*

| Threshold coefficients | | |
| --- | --- | --- |
| KSS scale | Estimate | 95% CI |
| 1\|2 | -3.88 | -5.87 – -1.91 |
| 2\|3 | -1.81 | -2.61 – -1.01 |
| 3\|4 | -0.51 | -1.08–0.07 |
| 4\|5 | 0.48 | -0.10–1.05 |
| 5\|6 | 1.26 | 0.61–1.90 |
| 6\|7 | 2.53 | 1.71–3.35 |
| 7\|8 | 3.83 | 2.87–4.79 |
| 8\|9 | 5.70 | 4.38–7.01 |
